# Editorial: Impact of ketogenic diet on metabolic and brain health

**DOI:** 10.3389/fnins.2022.1107741

**Published:** 2023-01-09

**Authors:** Paola Sacchetti, Shalini Jain, Hariom Yadav, Antonio Paoli

**Affiliations:** ^1^Department of Biology, University of Hartford, West Hartford, CT, United States; ^2^Department of Neurosurgery and Brain Repair, University of South Florida, Tampa, FL, United States; ^3^USF Center for Microbiome Research, Department of Neurosurgery and Brain Repair, University of South Florida, Tampa, FL, United States; ^4^Department of Biomedical Sciences, University of Padua, Padua, Italy

**Keywords:** cognitive function, microbiome and dysbiosis, inflammation, diet, metabolic therapy, Alzheimer's disease, neuroinflammation

In today's society, good eating habits and dietary interventions are publicized and advised for all conditions, from weight loss to cardiovascular health, to cancer and cognitive impairments. However, evidence of measurable benefits for these non-pharmacological interventions is limited and hinders their prescription and implementation as adjuvant therapies. The current Research Topic aimed at collecting evidence to support the usage of ketotherapies (KTs) as interventions to improve brain and metabolic health.

The ketogenic diet (KD) is a high fat and low carbohydrate diet originally developed to treat refractory epilepsy by mimicking the biochemical phenomenon that ensues during fasting. Its popularity has recently risen, and it is used worldwide to treat epilepsy, promote weight loss, and address diabetes. The standard KD comes in 4:1 ratio of fat to carbohydrates plus proteins, and variations of this diet are increasingly available to enhance compliance. The KD is a complex dietary regime; thus, defining its mechanisms of action is challenging. However, it redirects the standard energy source from carbohydrates to ketone bodies. In a balanced diet, glucose is the brain's primary energy source; however, in some neurological conditions brain cells have difficulties metabolizing efficiently glucose. In such conditions, KTs may help by boosting the production of ketone bodies, which can be efficiently utilized by brain cells.

As KTs have been shown to influence metabolism, inflammation, gut microbiome and neurological conditions (Masino, 2022), the goal of this Research Topic was to tackle the interplay between these domains to identify KT-dependent mechanisms that have positive impact on cognitive functions.

Carbohydrate-restricted diets are successful weight-loss measures that seem beneficial also in addressing metabolic syndromes, such as diabetes and obesity. In fact, these diets affect abdominal circumference and reduce blood glucose, while improving triglycerides and HDL cholesterol. Interestingly, abdominal adiposity has been linked to cognitive deficiencies both in mice and humans (Salas-Venegas et al., 2022) and addressing this condition brings to the frontline therapies that can target simultaneously metabolic dysfunctions and cognitive decline. Within this Research Topic, Kackley et al. comprehensively described the relation between weight-loss, KTs and treatment compliance, by evaluating the impact of ketone salt supplements on mood, which can be negatively impacted during the initial KD implementation.

As metabolic syndromes are intrinsically linked to chronic systemic inflammation, interventions such as KTs, that improve glycemic response and insulin sensitivity (Yuan et al., 2020), should also improve systemic inflammation. Recent findings support the role of a KD in modulating gut Th17 cells' activity, which in turn promotes metabolic syndrome-associated inflammatory phenotypes (Fabbrini et al., 2013; Dalmas et al., 2014), as well as protection from obesity and metabolic syndrome (Garidou et al., 2015; Hong et al., 2017). Therefore, the impact of diet and the gut microbiota on Th17 cells may be beneficial or detrimental depending on the context. In overweight subjects, Ang and colleagues (Ang et al., 2020) demonstrated a gut microbiota-mediated reduction of pro-inflammatory Th17 cells after 4 weeks -on a KD. On the contrary, Kawano and coworkers (Kawano et al., 2022) demonstrated that gut microbiota is necessary to maintain the Th17 role on weight and glycemic control. In general, the innate inflammasome pathway seems to be modulated through the different effects of the KD (glucose reduction, the specific activity of BHB on different inflammation pathways and others) (Rutsch et al., 2020). Considering the well-demonstrated role of microbiota on gut inflammation and the positive effects of the KD on the latter (Paoli et al., 2019), the utilization of the KD to treat different neuroinflammatory conditions such as multiple sclerosis, Alzheimer's disease (AD), Parkinson's disease, and mood disorders should be taken into account. The brain may be considered an immune-privileged organ due to the role of the Blood Brain Barrier as a control checkpoint that prevents the access of immune cells and immune mediators to the brain. However, the relationship between the KD, the immune system, the brain, and the gut is complex and not yet fully understood. In this Research Topic, Robbins and Solito performed a systematic evaluation of the effects of caloric restriction and KD on neuropsychiatric diseases and pointed to the modulation of neuroinflammation to explain diet-related cognitive changes. In terms of neurodegeneration, the real benefit would result from interventions controlling inflammation, but also directly affecting the hallmarks of disease progression. This hypothesis was described by Brinkley et al. in their study presented here. The results suggest that KT-induced improvements in localized adiposity are correlated to changes in circulating biomarkers of mild cognitive impairment in prediabetics at risk of AD.

Although the brain is highly dependent on glucose, during glucose deprivation it can efficiently metabolize ketone bodies, even during aging (Castellano et al., 2019). Bioenergetics and glucose metabolism dysfunctions have been identified in neurological diseases. In such circumstances, the availability of circulating ketone bodies might provide an alternative energy supply to maintain or regain cognitive functions. AD has a multifactorial pathogenesis of hypometabolism, neuroinflammation, gut dysbiosis and cognitive alterations and is a candidate disorder for testing the efficacy of KTs. Evidence shows that ketonic conditions can improve cognitive functions in animal models and patients with MCI and AD (Altayyar et al., 2022). Yet, the diverse regimens used in clinical and preclinical experiments confine the overall results and make it difficult to identify efficacious parameters to translate from bench to bedside. Moreover, the physiological and molecular mechanisms that produce improvements remain elusive, impeding the appropriate targeting of specific symptoms and diseases. In this context, by reviewing mainly preclinical studies, Taylor et al. build a case for the beneficial role of KTs in decreasing the accumulation of Amyloid β, a marker of preclinical AD. They propose converging mechanisms that could explain mechanistically how these therapies could slow Amyloid β accumulation and be beneficial for patients.

## Conclusion

The current Research Topic showcases KTs as effective tools to improve mood, adiposity, neuroinflammation and affect levels of disease hallmarks. Although causality remains elusive for KT-direct effects on cognition, most evidence points to strong anti-inflammatory and metabolic effects due to gut microbiota changes that might confer neuroprotection. Given the emerging role of the gut-brain-microbiome axis, if KD can modulate inflammation, gut- and metabolic- health, which all positively impact cognition, therefore KTs should be beneficial adjuvant treatments to ameliorate cognitive functions.

## Author contributions

PS contributed to the conception of the editorial. PS, HY, and AP contributed to the design. PS, SJ, and AP wrote sections of the manuscript. All authors contributed to manuscript revision, read, and approved the submitted version.
